# Biventricular Interaction During Acute Left Ventricular Ischemia in Mice: A Combined In-Vivo and In-Silico Approach

**DOI:** 10.1007/s10439-023-03293-z

**Published:** 2023-07-15

**Authors:** M. J. Colebank, R. Taylor, T. A. Hacker, N. C. Chesler

**Affiliations:** 1 Edwards Lifesciences Foundation Cardiovascular Innovation and Research Center, and Department of Biomedical Engineering, University of California, Irvine, Irvine, CA USA; 2 Cardiovascular Research Center, University of Wisconsin-Madison, Madison, WI USA

**Keywords:** Computational model, Parameter estimation, Myocardial infarction, Biventricular interaction, Sensitivity analysis, Multiscale modeling

## Abstract

Computational models provide an efficient paradigm for integrating and linking multiple spatial and temporal scales. However, these models are difficult to parameterize and match to experimental data. Recent advances in both data collection and model analyses have helped overcome this limitation. Here, we combine a multiscale, biventricular interaction model with mouse data before and after left ventricular (LV) ischemia. Sensitivity analyses are used to identify the most influential parameters on pressure and volume predictions. The subset of influential model parameters are calibrated to biventricular pressure–volume loop data (*n* = 3) at baseline. Each mouse underwent left anterior descending coronary artery ligation, during which changes in fractional shortening and RV pressure–volume dynamics were recorded. Using the calibrated model, we simulate acute LV ischemia and contrast outputs at baseline and in simulated ischemia. Our baseline simulations align with the LV and RV data, and our predictions during ischemia complement recorded RV data and prior studies on LV function during myocardial infarction. We show that a model with both biventricular mechanical interaction and systems-level cardiovascular dynamics can quantitatively reproduce in-vivo data and qualitatively match prior findings from animal studies on LV ischemia.

**Supplementary Information:**

The online version contains supplementary material available at 10.1007/s10439-023-03293-z.

## Introduction

Coronary artery disease, which can lead to myocardial infarction, accounts for roughly 41% of all cardiovascular-related deaths [33]. Acutely disrupted blood flow and oxygen supply to the myocardium causes cell death and systolic dysfunction, raising diastolic ventricular and atrial filling volumes [3]. Increases in left ventricular (LV) volume raise left atrial and pulmonary venous pressure [9], the latter of which is hypothesized to initiate vascular remodeling and pulmonary hypertension with the eventual consequence of right heart failure [1, 26]. This cascade of events is difficult to integrate from experimental or clinical data alone. A better understanding of the acute and systems-level effects of LV ischemia will provide insight into the initiators of long-term cardiac remodeling. Moreover, an analysis of cardiovascular biomechanics after recent myocardial infarction may reveal contributors to long-term cardiac failure and comorbidities such as pulmonary hypertension.

The right ventricle (RV) is mechanically linked to the LV through the interventricular septum (S). Previous canine studies [7] in the absence of RV electrical pacing reported that 68% of RV systolic pressure and 80% of pulmonary flow output were attributed to LV and S contributions. Follow-up investigations [12] also reported that RV ischemia reduced pulmonary systolic pressures by 4 mmHg, while septal ischemia had a greater effect on the RV and reduced pulmonary systolic pressures by 8 mmHg. Thus, systolic dysfunction in either chamber impairs whole heart function, drawing on the importance of biventricular interaction under pathophysiological conditions.

In-vivo experiments provide insightful but isolated measurements of cardiovascular function. In-silico computational models can integrate multimodal data (e.g., pressure, imaging, and other measurements) from in-vivo experiments to characterize a subject’s hemodynamic state. These models can also test mechanistic hypotheses surrounding disease progression. For example, early work using isolated ventricular elastance models in a closed loop compartment model investigated the link between LV systolic dysfunction and pulmonary venous pressure [4]. While reduced LV end-systolic elastance alone could not replicate the rise in pulmonary venous pressure seen clinically, additional increased systemic venous volume and pericardial constraints in the model framework could recreate these established findings. Efforts have also resulted in the incorporation of LV remodeling and hemodynamic reflexes [37], which synergistically contribute to LV remodeling.

These prior computational studies did not explicitly account for biventricular interaction or include multiscale mechanisms. The cutting-edge reduced order model of ventricular interaction is the three-segment (“TriSeg”) model by Lumens et al., which represents the LV, RV, and S as thick walled, spherical chambers driven by myocyte dynamics [18]. Several authors have had success in using this framework to simulate diseases such as pulmonary hypertension [32] and LV ischemia [16]. These models contain numerous parameters, requiring a formal model analysis to determine which parameters are influential and identifiable given limited data [5]. A proper analysis of the model and experimental design is tedious; however, identifying model sensitivity to the parameters as well as the uncertainty in parameter estimates and model outputs is crucial for drawing conclusions from the model itself. The combination of multiscale in-silico models, robust parameter estimation from in-vivo data, and proper uncertainty quantification is necessary as computer models begin to be used for clinical analyses [6].

Here, we combine our previously reported multiscale model [5] with data from a cohort of male mice in baseline and acutely ischemic conditions. Echocardiographic, pressure, and volume data from the LV, RV, and systemic arteries are collected pre-ischemia. We determine a subset of influential parameters using sensitivity analyses and calibrate the multiscale model to baseline data. We compare our model simulations and their uncertainty to the measured data pre- and post-ischemia and provide predictions of strain and left atrial pressure to investigate acute changes in cardiac biomechanics during LV ischemia [19].

## Methods

### In-Vivo Animal Data

**Fig. 1:**
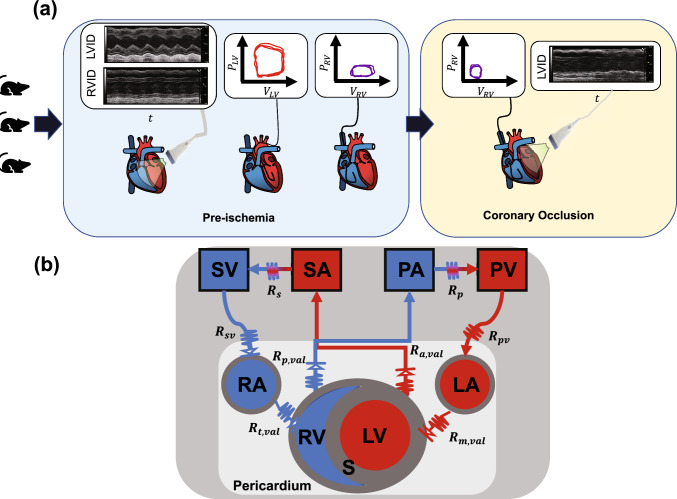
Experimental and model schematics. **a** Three male mice underwent non-invasive echocardiography, providing measurements of ventricular inner diameter. A pressure–volume catheter was then placed in the LV chamber, data were recorded, the catheter was removed, and placed in the RV. While the RV catheter was still in, the left anterior descending coronary artery was ligated, and RV pressure–volume data were recorded. Echocardiography was repeated. **b** Schematic of the closed loop computational model. The two ventricles are coupled through a dynamic septal wall using the TriSeg framework. All four heart chambers are encased in a passive, pericardial sack and connected to compliant arterials and venous compartments. Resistors connect all compartment model components. *LA* left atrium, *LV* left ventricle, *PA* pulmonary arteries, *PV* pulmonary veins, *RA* right atrium, *RV* right ventricle, *S* septum, *SA* systemic arteries, *SV* systemic veins

All animal procedures were approved by the University of Wisconsin-Madison Institutional Animal Care and Use Committee. Three adult C57/B16 male mice (20–22 weeks old) were anaesthetized with 5% isoflurane and maintained with 1–2% isoflurane and room air throughout all procedures. Mice were put on a heated platform to maintain a body temperature of 37 °C and measure ECG activity. Transthoracic echocardiography (Vevo 3100, Visual Sonics) was used to identify systolic and diastolic inner diameter and fractional shortening for both the LV and RV. A cutdown was performed on the right carotid artery and a 1.2 Fr pressure catheter (Transonic) was placed and advanced to the ascending aorta to measure systemic pressures. Finally, the thoracic cavity was entered, and the heart was exposed. A 1.2 Fr pressure–volume catheter with 4.0 mm spacing (Transonic) was inserted into the LV via direct stick through the myocardial wall. Baseline systemic and LV data were recorded. The catheter was removed and a second 1.2 Fr pressure–volume catheter with 3.5 mm spacing (Transonic) was place in the RV free wall aligned with the pulmonary valve. Baseline systemic and RV data were collected. A 7-0 suture was placed around the left anterior coronary artery mid-ventricle and tied while still recording RV data. Typical ECG changes and blanching were noted. Pressure and volume measurements were recorded at 500 Hz and analyzed on commercially available software (Notocord Systems, Croissy Sur Seine, France). Then, the mice were sacrificed and the four heart chambers were dissected and weighed [26]. Heart chamber weight is converted to wall volume using a constant density of 1.053 g/cm^3^ and used in the computational model described later. We assume the septum occupies 1/3 of the LV volume [21]. A schematic of the experimental design is provided in Fig. 1a.

We use the MATLAB (Mathworks, Natick, MA) Gaussian smoothing filter to smooth pressure–volume signals. We use a smoothing factor of 0.05, corresponding to 11-45 data point smoothing depending on signal length. We visually inspected signals to ensure systolic, diastolic, and general waveform shape were maintained. In-house algorithms were used to separate signals into beat-by-beat datasets for analyses. To account for discrepancies in pressure–volume phase due to catheter placement, volume traces were slightly shifted to ensure maximal chamber volume occurred at the upstroke of ventricular pressure. We use a heartbeat averaged waveform from each animal and cardiovascular component when performing model calibration and uncertainty quantification.

### Mathematical Model

We use a previously developed multiscale cardiovascular model [16, 18]. The model components include (1) a modified Hill model of sarcomere shortening, (2) an empirical model of cardiomyocyte calcium handling, (3) four spherical cardiac chambers including biventricular interaction, and (4) a zero-dimensional (0D) hemodynamics model. All computations are performed in units of kPa, cm (or μm in the sarcomere), s, and mL and subsequently converted back to mmHg, mm, and μL for comparison to the measured data.

The sarcomere length $L_{\text{s}}$ (μm) is determined from the myofiber strain, $\varepsilon_{\text{f}}$ within each chamber

1 $$L_{\text{s}} = L_{\text{s,ref}} \exp \left( {\varepsilon_{\text{f}} } \right),$$

where $L_{\text{s,ref}} = 2.0$ (μm) is the reference sarcomere length at zero strain (i.e., $\varepsilon_{\text{f}} = 0$) given by equation (S2) in the supplement. The contractile sarcomere element has length $L_{\text{sc}}$ (μm) and is in series with an elastic series element with length $L_{\text{se}} = L_{\text{s}} - L_{\text{sc}}$ (μm). Sarcomere shortening is described by

2 $$\frac{{dL_{\text{sc}} }}{dt} = \left( {\frac{{L_{\text{s}} - L_{\text{sc}} }}{{L_{\text{s,iso}} }} - 1} \right)v_{0} ,$$

where $L_{\text{s,iso}}$ (μm) is the elastic series element length in an isometrically stressed state, and $v_{0}$ (μm/s) is the velocity of sarcomere shortening with zero load [18]. Sarcomere activation is modeled as the sum of rise and decay terms

3 $${\Psi }_{\text{rise}} = \frac{1}{{\tau_{\text{rise}} }}C_{\text{L}} \left( {L_{\text{sc}} } \right)F_{\text{rise}} , \quad C_{\text{L}} \left( {L_{\text{sc}} } \right) = \tanh \left( {4\left( {L_{\text{sc}} - L_{{\text{sc}},0} } \right)^{2} } \right),$$

where $C_{\text{L}}$ (dimensionless) represents the increase in contractility with sarcomere length and $L_{{\text{sc}},0}$ (μm) represents the contractile element length with zero active stress. The second term $F_{\text{rise}}$ (dimensionless) describes changes in cardiomyocyte intracellular calcium

4 $$F_{\text{rise}} \left( t \right) = 0.02x^{3} \left( {8 - x} \right)^{2}\exp \left( { - x} \right) ,\quad x = \min\left( {8,\left( {t/\tau_{\text{rise}} } \right)} \right),$$

where $\tau_{\text{rise}}$ (s) scales the rise in contractility. Calcium decay is given by

5 $${\Psi }_{\text{decay}} = \frac{1}{{\tau_{\text{decay}} }}\left( {\frac{{{\Gamma }_{\text{rest}} - {\Gamma }\left( t \right)}}{{1 + \exp \left( {\left( {T\left( {L_{\text{sc}} } \right) - t} \right)/\tau_{\text{decay}} } \right) }}} \right), \quad T\left( {L_{\text{sc}} } \right) = \tau_{\text{sys}} \left( {0.29 + 0.3L_{\text{sc}} } \right).$$

The decay in activation saturates at the diastolic value ${\Gamma }_{\text{rest}}$ (dimensionless), and depends on the systolic contraction and diastolic decay parameters $\tau_{\text{sys}}$ and $\tau_{\text{decay}}$ (s), respectively. Equations (3) and (5) dictate the total contractile state, ${\Gamma }$ (dimensionless), which is modeled by the differential equation:

6 $$\frac{{{\text{d}}{\Gamma }}}{{\text{d}}t} = {\Psi }_{\text{rise}} + {\Psi }_{\text{decay}} .$$

The active stress, $G_{\text{act}}$ (kPa) is finally calculated as

7 $$G_{\text{act}} = \sigma_{\text{act}} {\Gamma } \left( {L_{\text{sc}} - L_{{\text{sc}},0} } \right)\left( {\frac{{L_{\text{s}} - L_{\text{sc}} }}{{L_{\text{se,iso}} }}} \right),$$

where $\sigma_{\text{act}}$ (kPa) is a scaling parameter [18]. Passive sarcomere stretch is relative to the passive reference length $L_{\text{s,pas,ref}}$ (μm)

8 $$\lambda_{\text{s,pas}} = \frac{{L_{\text{s,ref}} }}{{L_{\text{s,pas,ref}} }}\exp \left( {\varepsilon_{\text{f}} } \right) .$$

The passive stresses are separated into those attributed to the extracellular matrix (ECM) and Titin

9 $$G_{\text{ECM}} = \sigma_{\text{ECM}} \left( {\lambda_{\text{s,pas}}^{{k_{\text{ECM}} }} - 1} \right), \quad G_{\text{Titin}} = \sigma_{\text{Titin}} \left( {\lambda_{\text{s,pas}}^{{k_{\text{Titin}} }} - 1} \right),$$

where $\sigma_{\text{ECM}}$ and $\sigma_{\text{Titin}}$ (kPa) are scaling parameters and $k_{\text{ECM}}$ and $k_{\text{Titin}}$ (dimensionless) account for non-linear chamber stiffening [34].

The sarcomere model is embedded within each cardiac chamber and the interventricular septum. Ventricular interaction across the septal wall is prescribed using the TriSeg model [18]. Cardiac chamber geometries are modeled as spherical structures described by a mid-wall volume $V_{\text{m}}$ (cm^3^), mid-wall curvature $C_{\text{m}}$ (1/cm), and mid-wall cross-sectional area $A_{\text{m}}$ (cm^2^) and parameterized by a reference mid-wall area, $A_{\text{m,ref}}$ (cm^2^), and a wall volume, $V_{wall}$ (cm^3^). Tension balance across the LV, RV, and S walls are enforced by two algebraic constraints. Details regarding the chamber equations can be found in the Supplementary Material. All four heart chambers are enclosed in a pericardium. We assume that the pericardial sack has a reference volume, $V_{0,{\text{peri}}}$, and exhibits a non-linear pressure–volume relationship driven by total blood volume in the heart [15]. Pericardial pressure, $p_{\text{peri}}$ (kPa), is then

10 $$p_{\text{peri}} = \exp \left( {k_{\text{peri}} \left( {\frac{{V_{heart} }}{{V_{0,{\text{peri}}} }} - 1} \right)} \right),$$

where $V_{heart}$ (μL) represents the total volume in all four heart chambers and $k_{\text{peri}}$ (kPa) describes the exponential rise in pericardial pressure. This pressure value is added to each cardiac chamber as an external pressure source.

Arteries and veins are modeled as compliant compartments. Changes in blood volume $V$ (mL), flow $q$ (mL/s), and pressure $p$ (kPa) are described as [6]

11 $$\frac{{\text{d}}V}{{{\text{d}}t}} = q_{in} - q_{out} ,$$

12 $$p = \frac{{\left( {V - V_{un} } \right) }}{C},$$

13 $$q = \frac{{p_{out} - p_{in} }}{R},$$

where $V_{un}$ ($m$L) is the unstressed volume, $C$ ($m$L / kPa) is the vascular compliance, and $R$ (kPa s /$m$L) is the vascular resistance between compartments. Cardiac valves are modeled as diodes and are only open when the pressure gradients are positive. We also include a valve between the systemic veins and the right atrium, which prevents backflow and can mimic vena cava collapse. A schematic of all model components can be found in Fig. 1(b).

## Model Analysis

The mathematical model includes 18 differential equations (eight compartment volumes, $V\left( t \right)$, five sarcomere states, $L_{\text{sc}} \left( t \right),$ and five contractility states, ${\Gamma }\left( t \right)$) as well as two equilibria constraints (tension balance for the TriSeg model, see Supplemental Material). These equations require a total of 53 parameters, described in Table 1, which cannot be inferred simultaneously. Several of the parameters in the sarcomere model, such as the various reference sarcomere lengths, are fixed to values consistently used in the literature [18, 34]. We calculate wall volumes, $V_{wall}$, as the ratio of chamber mass to myocardial density, 1.053 g/cm^3^. [5]This leaves 38 free parameters to analyze by Morris screening and local sensitivity analysis [5, 23]. Table 1 and the Supplementary Material describe how nominal parameters are calculated.

**Table 1:** Model parameters

Parameter	Description	Units	Nominal value/equation
*Sarcomere parameters*			
$L_{{\text{s,ref}},j}$*	Reference sarcomere length at zero strain	μm	$\left( {2.0} \right), 2.0$
$L_{\text{s,iso}}$*	Elastic series element length in isometric state	μm	$\left( {0.04} \right),0.04$
$L_{\text{s,pas,ref}}$*	Reference length for passive all constituents	μm	$\left( {1.8} \right),1.8$
$v_{0,j}$	Velocity of sarcomere shortening	μm/s	$\left( {24} \right), 12$
$L_{{\text{sc}},0}$*	Contractile element length	μm	$\left( {1.51} \right),1.51$
${\Gamma }_{\text{rest}}$*	Resting contractility	Dimensionless	$\left( {0.02} \right),0.02$
$\tau_{{\text{rise}},j}$	Rise in contractility scaling	s	$\left( {0.0375 \cdot T} \right)$, $0.009$
$\tau_{\text{{decay}},j}$	Decay in contractility scaling	s	$\left( {0.005 \cdot T} \right), 0.009$
$\tau_{\text{{sys}},j}$	Length of systole	s	$\left( {0.15 \cdot T} \right),0.038$
$\tau_{\text{{offset}},A}$	Offset of atrial systole	s	$0.18 \cdot T$
$k_{\text{{ECM}},j}$	Non-linear ECM stiffness exponent	Dimensionless	$\left( {10} \right),10$
$k_{{\text{Titin}},j}$	Non-linear Titin stiffness exponent	Dimensionless	$\left( 6 \right),6$
$\sigma_{\text{{ECM}},j}$	Passive ECM stress scaling factor	kPa	$\left( {0.08} \right),0.08$
$\sigma_{{\text{Titin}},j}$	Passive Titin stress scaling factor	kPa	$\left( {0.25} \right),0.25$
$\sigma_{act,j}$	Active stress scaling factor	kPa	$\left( {35} \right), 75$
*TriSeg/cardiac parameters*			
$V_{\text{LA,wall}}$*	LA wall volume	cm^3^	Chamber mass/$1.053\,{\text{g/cm}}^{3}$
$V_{\text{LV,wall}}$*	LV wall volume	cm^3^	Chamber mass/$1.053\,{\text{g/cm}}^{3}$
$V_{\text{RA,wall}}$*	RA wall volume	cm^3^	Chamber mass/$1.053\,{\text{g/cm}}^{3}$
$V_{\text{RV,wall}}$*	RV wall volume	cm^3^	Chamber mass/$1.053\,{\text{g/cm}}^{3}$
$V_{\text{S,wall}}$*	S wall volume	cm^3^	Chamber mass/$1.053\,{\text{g/cm}}^{3}$
$A_{\text{m,ref,LA}}$	LA reference area	cm^2^	$\left[ {0.15,0.15,0.15} \right]$
$A_{\text{m,ref,LV}}$	LV reference area	cm^2^	$\left[ {0.50,0.70,0.50} \right]$
$A_{\text{m,ref,RA}}$	RA reference area	cm^2^	$\left[ {0.15,0.15,0.15} \right]$
$A_{\text{m,ref,RV}}$	RV reference area	cm^2^	$\left[ {0.55,0.80,0.50} \right]$
$A_{\text{m,ref,S}}$	S reference area	cm^2^	$\left[ {0.25,0.35,0.35} \right]$
$V_{0,{\text{peri}}}$	Reference volume for pericardial space	mL	$\left[ {0.159,0.178,0.165} \right]$
$k_{\text{peri}}$	Material parameter of pericardial tissue	kPa	$10$
*Cardiovascular system parameters*			
$R_{\text{a,val}}$	Aortic valve resistance	kPas/mL	0.1/$CO$
$R_{\text{m,val}}$	Mitral valve resistance	kPa s/mL	0.75/$CO$
$R_{\text{p,val}}$	Pulmonic valve resistance	kPa s/mL	0.1/$CO$
$R_{\text{t,val}}$	Tricuspid valve resistance	kPa s/mL	0.75/$CO$
$R_{\text{vc}}$	Vena Cava resistance	kPa s/mL	$\left( {\overline{P}_{\text{sv}} - P_{\text{ra,min}} } \right)/CO$
$R_{\text{pv}}$	Pulmonary venous resistance	kPa s/mL	$\left( {\overline{P}_{\text{pv}} - P_{\text{la,min}} } \right)/CO$
$R_{\text{sys}}$	Systemic circulation resistance	kPa s/mL	$\left( {P_{\text{sa,max}} - P_{\text{sys,cap}} ,} \right) / CO$
$R_{\text{pulm}}$	Pulmonary circulation resistance	kPa s/mL	$\left( {P_{\text{pa,max}} - P_{\text{pulm,cap}} } \right)/CO$
$C_{\text{sa}}$	Compliance of systemic arteries	mL/kPa	$V_{\text{sa}} /P_{\text{sa,max}}$
$C_{\text{sv}}$	Compliance of systemic veins	mL/kPa	$V_{\text{sv}} /\overline{P}_{\text{sv}}$
$C_{\text{pa}}$	Compliance of pulmonary arteries	mL/kPa	$V_{\text{pa}} /P_{\text{pa,max}}$
$C_{\text{pv}}$	Compliance of pulmonary veins	mL/kPa	$V_{\text{pv}} /\overline{P}_{\text{pv}}$

Parameters denoted with an * are fixed before performing Morris screening or local sensitivity analyses. Cardiac parameters denoted with a subscript $j$ have an atrial and ventricular component, with atrial values provided in parenthesis. Mouse-specific values are given in square brackets. $T$ (s) represents the mouse-specific average cardiac cycle length. Pressure and volume variables are described in detail in the Supplementary Material

*Cap* capillary, *CO* cardiac output, *LA* left atrium, *LV* left ventricle, *PA* pulmonary arteries, *PV* pulmonary veins, *RA* right atrium, *RV* right ventricle, *S* septum, *SA* systemic arteries, *SV* systemic veins

Morris screening is an efficient screening tool that uses coarse approximations of model sensitivity to determine which parameters are non-influential [23]. We use simulated LV and RV pressure–volume relationships as well as systemic arterial pressure as our quantities of interests. We rank parameter importance based on the modified sample mean, $\mu^{*}$, and sample variance, $s^{2}$, through the index ${\mathcal{M}} = \sqrt {\mu^{*2} + s^{2} }$ [5, 36]. Similar to van Osta et al. [23], parameters consistently less influential than the mean value of ${\mathcal{M}}$ on all five outputs are deemed non-influential and fixed. Parameter bounds for sampling are set at $\pm 20\%$ from the nominal value for each mouse.

Though Morris screening can identify the least influential parameters, it does not provide detailed information about parameter interactions, nor does it provide information about identifiability. Local sensitivity analysis can provide approximate metrics of local identifiability [22]. The local sensitivity of each pressure or volume, denoted as $f\left( {t;{\varvec{\theta}}} \right)$, is approximated by centered finite differences

14 $$S_{i} = \frac{{df\left( {t;{\varvec{\theta}}} \right)}}{{d\theta_{i} }} = \frac{{f\left( {t;{\varvec{\theta}} + h{\varvec{e}}_{{\varvec{i}}} } \right) - f\left( {t;{\varvec{\theta}} - h{\varvec{e}}_{i} } \right)}}{2h},$$

where $h = 0.01$ is the step size and ${\varvec{e}}_{i}$ is the unit vector in the ith direction. To account for differences in parameter and output magnitudes, we use dimensionless sensitivities by multiplying by $\theta_{i} /f\left( {t;{\varvec{\theta}}} \right)$ [22]. We use the local sensitivity vectors to construct an approximate Fisher information matrix, ${\varvec{F}} = {\varvec{S}}^{ \top } {\varvec{S}}$, and assess practical identifiability in an asymptotic sense [6, 10]. If ${\varvec{F}}$ is ill-conditioned, then the parameter subset is deemed non-identifiable and requires reduction. In this study, we investigate the local sensitivity of the reduced parameter subset after Morris screening. If $F$ is ill-conditioned, the least influential parameter is fixed and the algorithm is iterated again. We continue this scheme until ${\text{cond}}\left( {\varvec{F}} \right) \le 10^{5}$, which is our numerical ill-conditioning cutoff.

### Parameter Inference and Uncertainty Quantification

The reduced subset is calibrated to data using non-linear, weighted least squares [6]. We minimize the negative log-likelihood, $- LL$, defined by

15 $$- LL\left( {\varvec{\theta}} \right) = \frac{N}{2}\log \left( {2\pi det\left({\varvec{\Sigma}}\right)} \right) + \frac{1}{2}\left[ {\left( {{\varvec{y}}^{data} - f\left( {{\varvec{t}};{\varvec{\theta}}} \right)} \right)^{ \top } {{\varvec{\Sigma}}}^{ - 1} \left( {{\varvec{y}}^{data} - f\left( {{\varvec{t}};{\varvec{\theta}}} \right)} \right)} \right],$$

where ${\varvec{y}}^{data} = \, \left[ {{\varvec{p}}_{RV}^{data} ,{\varvec{V}}_{RV}^{data} ,{\varvec{p}}_{LV}^{data} ,{\varvec{V}}_{LV}^{data} ,{\varvec{p}}_{SA}^{data} } \right]$ denotes the measured data in the RV, LV, and systemic arteries (SA), $f\left( {t;{\varvec{\theta}}} \right)$ is the corresponding model output, and $N$ represents the number of data points, respectively. We infer the natural log transferred parameters to ensure that they have similar magnitudes. To account for the possible heteroskedastic error variance in the signal across the five measurement locations, we include a diagonal error covariance matrix, ${{\varvec{\Sigma}}} = {\text{diag}}\left( {\sigma_{i}^{2} } \right)$, which is updated using iteratively reweighted least squares (see the Supplemental Material) [30]. This error covariance includes five possibly unique error variances, $\sigma_{i}^{2}$, which correspond to the RV, LV, and SA measurements. Equation (15) is updated and minimized using *fminunc* in MATLAB and halted once the estimates of ${\varvec{\theta}}$ have converged.

The inferred log-scaled parameters, $\hat{\varvec{\theta }}$, and calibrated model response $\hat{\varvec{Y}}$, carry some uncertainty due to measurement error. We construct 95% confidence intervals about our inferred log parameters by calculating [2, 30]

16 $$\, \left[ {\widehat{{\theta_{i}^{ - } }},\widehat{{\theta_{i}^{ + } }}} \right] = \hat{\theta }_{i} \pm t_{{N_{tot} - N_{par} }}^{0.975} \sqrt[ ]{{{\mathbf{\mathcal{C}}}_{ii} }}, \, {\mathbf{\mathcal{C}}}_{ii} = \left( {\hat{\varvec{S}}^{ \top } \hat{\Sigma }^{ - 1} \hat{\varvec{S}} } \right)^{ - 1} .$$

The matrix ${\mathbf{\mathcal{C}}}_{\varvec{ }}$ is the asymptotic parameter variance–covariance matrix, $\hat{\Sigma }$ is the estimated diagonal error covariance calculated iteratively through equation (15), and $\hat{\varvec{S}}$ is the sensitivity of the log-likelihood at $\hat{\varvec{\theta }}$. The number of total data points $N_{tot}$ and the number of parameters $N_{par}$ are used to compute a two-sided t-score statistic $t_{{N_{tot} - N_{par} }}^{0.975}$ corresponding to a 95% confidence interval [2, 30]. We exponentiate the log-confidence intervals in equation (16) after computing all relevant indices.

The corresponding response confidence and prediction intervals around the optimal model output, $\hat{\varvec{Y}}$, are

17 $$\, \left[ {{\varvec{Y}}^{{CI_{ - } }} ,{\varvec{Y}}^{{CI_{ + } }} } \right] = \hat{\varvec{Y}} \pm t_{{N_{tot} - N_{par} }}^{0.975} \sqrt {\hat{\varvec{S}}^{ \top } {\mathbf{ \mathcal{C} }}\hat{\varvec{S}}}$$

and

18 $$\, \left[ {{\varvec{Y}}^{{PI_{ - } }} ,{\varvec{Y}}^{{PI_{ + } }} } \right] = \hat{\varvec{Y}} \pm t_{{N_{tot} - N_{par} }}^{0.975} \sqrt {{\hat{\mathbf{\Sigma }}} + \hat{\varvec{S}}^{{ \top }} {\mathbf{ \mathcal{C} }}\hat{\varvec{S}}} ,$$

where ${\hat{\mathbf{\Sigma }}}$ is the final estimate of the diagonal error covariance matrix. The confidence interval in equation (17) represents the uncertainty in the mean response without additional variation in the data, whereas the prediction interval from equation (18) also incorporates the measurement variance in new observations; hence, prediction intervals will be wider than their corresponding confidence intervals. Additional details can be found in the Supplemental Material.

### Simulated Myocardial Infarction

We simulate myocardial infarction by reducing LV active force at the sarcomere

19 $$F_{\text{rise}}^{\text{MI}} \left( t \right) = \gamma^{\text{MI}} \cdot F_{\text{rise}} \left( t \right),$$

where $\gamma^{\text{MI}}$ reflects the decrease in activation due to ischemia. We set $\gamma^{\text{MI}}$ such that LV ejection fraction is reduced by the same amount measured by echocardiography. We also examine changes in longitudinal wall strain

20 $$\lambda_{long} = \frac{{L_{\text{s}} \left( t \right) - L_{\text{s}}^{diastole} }}{{L_{\text{s}}^{diasole} }},$$

where $L_{\text{s}} \left( t \right)$ is the dynamic sarcomere length and $L_{\text{s}}^{diastole}$ is the length at end-diastole. The model and analysis scripts that compute the above are available on GitHub (https://github.com/mjcolebank/Colebank_2023_AcuteIschemia).

## Results

### In-Vivo Data

**Fig. 2:**
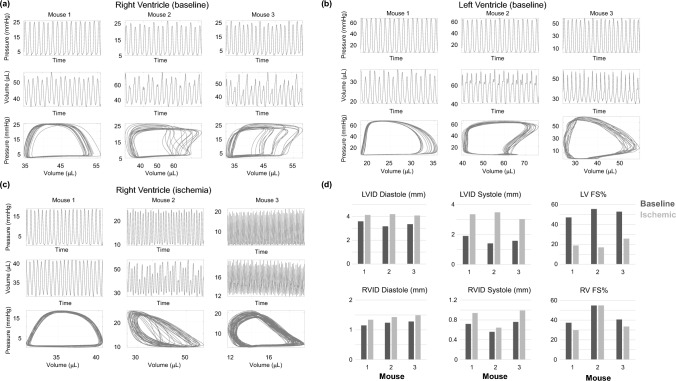
In-vivo data from three male mice. **a** Pressure, volume, and combined pressure–volume loops in the RV at baseline. **b** Pressure and volume data in the LV at baseline. **c** Pressure-volume data in the RV after left descending coronary artery ligation. **d** Baseline and ischemic echocardiography measurements in the LV and RV

Echocardiography and pressure–volume loops for each mouse are shown in Fig. 2. The time-dependent ventricular pressure and volume data are provided for each mouse, including RV pressure–volume data during ischemia in Fig. 2c. Ischemia introduces a decrease in RV volumes, especially in mouse 3. LV and RV inner diameters (Fig. 2d) are similar across all three mice. After coronary artery ligation, there is an increase in both systolic and diastolic LV inner diameter, contributing to a reduction in fractional shortening. There is also an increase in RV diastolic diameter, but not in RV systolic diameter nor in fractional shortening.

### Sensitivity Analyses

**Fig. 3:**
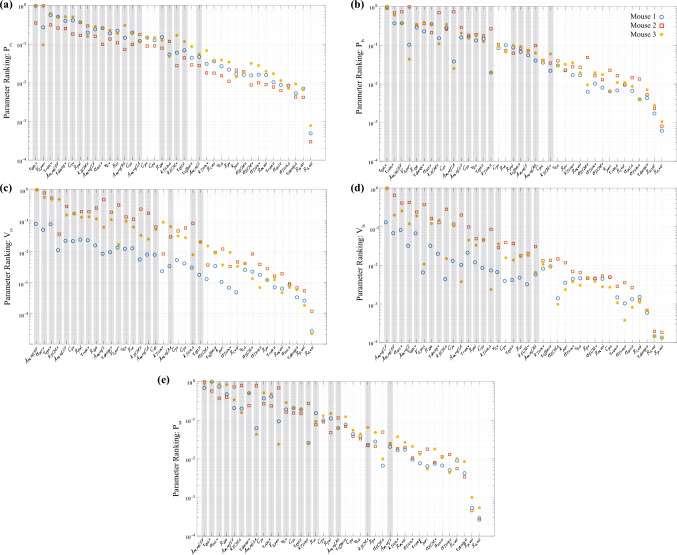
Parameter ranking using the combined index, ${\mathcal{M}} = \sqrt {\mu^{*2} + s^{2} }$, based on Morris screening for each mouse. Shaded columns represent the 20 parameters selected for additional analyses. **a** RV pressure. **b** LV pressure. **c** RV volume. **d** LV volume. **e** Systemic artery pressure. Each plot is normalized by the maximum index value for each mouse so that indices are scaled 0 to 1. Parameters deemed influential are shaded in gray across all five subplots

A total of 100 Morris screening initializations were run per mouse. Parameter ranking for the five different model outputs are provided in Fig. 3. The parameters describing the timing of ventricular systole and diastole ($\tau_{\text{rise,v}} ,\tau_{\text{decay,v}}$, and $\tau_{\text{sys,v}}$) are consistently the most influential on LV and RV pressure. The vascular parameters $R_{\text{sys}} , R_{\text{pulm}} ,$ and $C_{\text{sv}}$ are also influential on both pressure predictions. The LV, RV, and S reference areas are more influential on ventricular volume than ventricular pressure. Active force generation $\sigma_{\text{act,v}}$ and the reference pericardial volume $V_{0,{\text{peri}}}$ are moderately influential for all five outputs. Eighteen parameters have an average effect less than the mean, ${\overline{\mathcal{M}}},$ and are deemed non-influential.

The remaining 20-parameter subset is examined using local sensitivity analysis for each mouse. The matrix ${\varvec{F}}$ is invertible for all three mice but has a condition number between 1e7 and 1e8, which is numerically ill-conditioned by our criteria. Several iterations of subset reduction are carried out until ${\varvec{F}}$ has condition number below 1e5. A final subset using this approach consists of the 11 parameters

21 $${\varvec{\theta}}_{\text{opt}} = \, \left[ {A_{\text{m,ref,LV}} ,A_{\text{m,ref,RV}} ,A_{\text{m,ref,S}} ,\tau_{\text{rise,v}} ,\tau_{\text{decay,v}}, \tau_{\text{sys,v}} ,\sigma_{\text{act,v}} ,R_{\text{sys}} ,R_{pulm} ,C_{\text{sv}} ,V_{0,{\text{peri}}} } \right].$$

### Model Calibration and Uncertainty Quantification

**Fig. 4:**
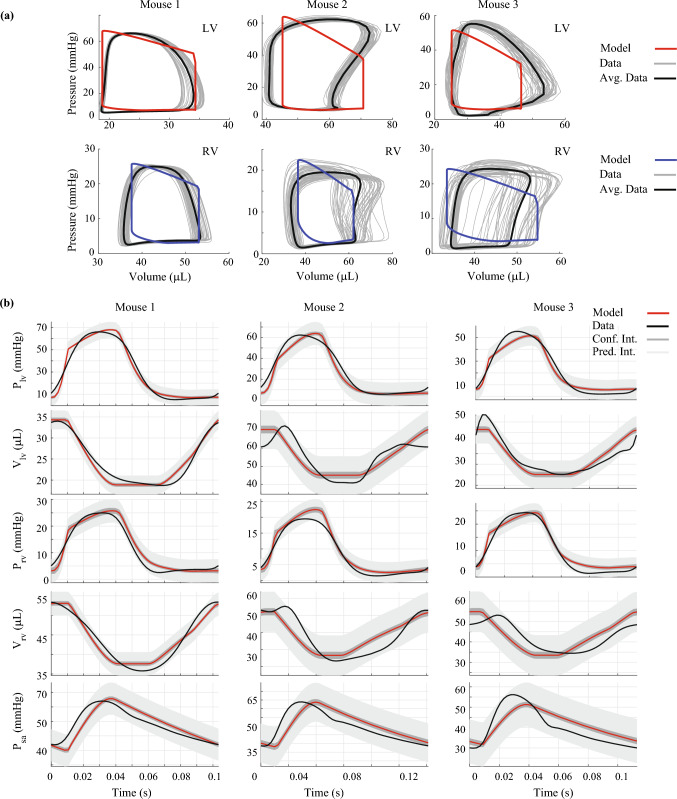
Comparison of calibrated model simulations with measured hemodynamic data. Pressure–volume loops in the LV and RV (**a**) for each mouse. Red and blue curves represent the calibrated model with the measured data, shown in gray. **b** shows optimal model solutions (red), confidence intervals (light gray), and predictions intervals (dark gray) for LV pressure, LV volume, RV pressure, RV volume, and SA pressure, respectively. Note that most of the beat-to-beat signals are captured within the uncertainty bounds

We infer $\widehat{{{\varvec{\theta}}_{ } }}$ for each mouse using the recorded baseline data. Optimal parameter estimates and the associated confidence intervals are provided in Table 2. Calibrated model pressure–volume loops, shown in Fig. 4a, align well with the recorded systolic and diastolic values. However, our calibrated simulations maintain the “ideal” pressure–volume loop shape while the data do not. Confidence and predictions intervals for the time-series model outputs are shown in Fig. 4b. The confidence intervals are more narrow than the corresponding predictions intervals, which contain nearly all the data across every measurement. Calibrated model simulations of pressure match well to the data, while the calibrated volume simulations show a slight discrepancy during isovolumic contraction. Parameter correlations at the optimal parameter value $\hat{\varvec{\theta }}$ can be found in the Supplementary Material along with the estimated error variances in ${\hat{\mathbf{\Sigma }}}$**.**

**Table 2:** Optimal parameter estimates and 95% confidence intervals

Parameter	Mouse 1	Mouse 2	Mouse 3
$A_{\text{m,ref,LV}}$ (cm^2^)	$0.483 \, \left[ {0.330,0.706} \right]$	$0.742 \, \left[ {0.557,0.987} \right]$	$0.549 \, \left[ {0.340,0.887} \right]$
$A_{\text{m,ref,RV}}$ (cm^2^)	$0.569 \, \left[ {0.435,0.744} \right]$	$0.785 \, \left[ {0.591,1.04} \right]$	$0.481 \, \left[ {0.368,0.629} \right]$
$A_{\text{m,ref,S}}$ (cm^2^)	$0.242 \, \left[ {0.108,0.543} \right]$	$0.250 \, \left[ {0.079,0.786} \right]$	$0.254 \, \left[ {0.098,0.658} \right]$
$\tau_{\text{rise,v}}$ (s)	$0.007 \, \left[ {0.006,0.009} \right]$	$0.009 \, \left[ {0.007,0.012} \right]$	$0.008 \, \left[ {0.006,0.010} \right]$
$\tau_{\text{decay,v}}$ (s)	$0.009 \, \left[ {0.008,0.010} \right]$	$0.009 \, \left[ {0.007,0.011} \right]$	$0.009 \, \left[ {0.007,0.011} \right]$
$\tau_{\text{sys,v}}$ (s)	$0. 032 \, \left[ {0.020,0.050} \right]$	$0.037 \, \left[ {0.025,0.054} \right]$	$0.034 \, \left[ {0.023,0.050} \right]$
$\sigma_{\text{act,v}}$ (kPa)	$72.3 \, \left[ {40.9,128} \right]$	$74.6 \, \left[ {25.9,215} \right]$	$77.6 \, \left[ {35.7,169} \right]$
$R_{\text{sys}}$ (kPa s/mL)	$41.8 \, \left[ {39.7, 44.0} \right]$	$25.2 \, \left[ {22.3,28.6} \right]$	$22.1 \, \left[ {19.6,24.9} \right]$
$R_{\text{pulm}}$ (kPa s/mL)	$10.2 \, \left[ {8.69,12.0} \right]$	$5.89 \, \left[ {4.49,7.73} \right]$	$7.34 \, \left[ {6.31,9.48} \right]$
$C_{\text{sv}}$ (mL/kPa)	$0.086 \, \left[ {0.076,0.099} \right]$	$0.095 \, \left[ {0.072,0.124} \right]$	$0.127 \, \left[ {0.106,0.152} \right]$
$V_{0,{\text{peri}}}$ (mL)	$0.149 \, \left[ {0.076,0.295} \right]$	$0.179 \, \left[ {0.148,0.216} \right]$	$0.162 \, \left[ {0.061,0.428} \right]$

### Simulated Myocardial Infarction

**Fig. 5:**
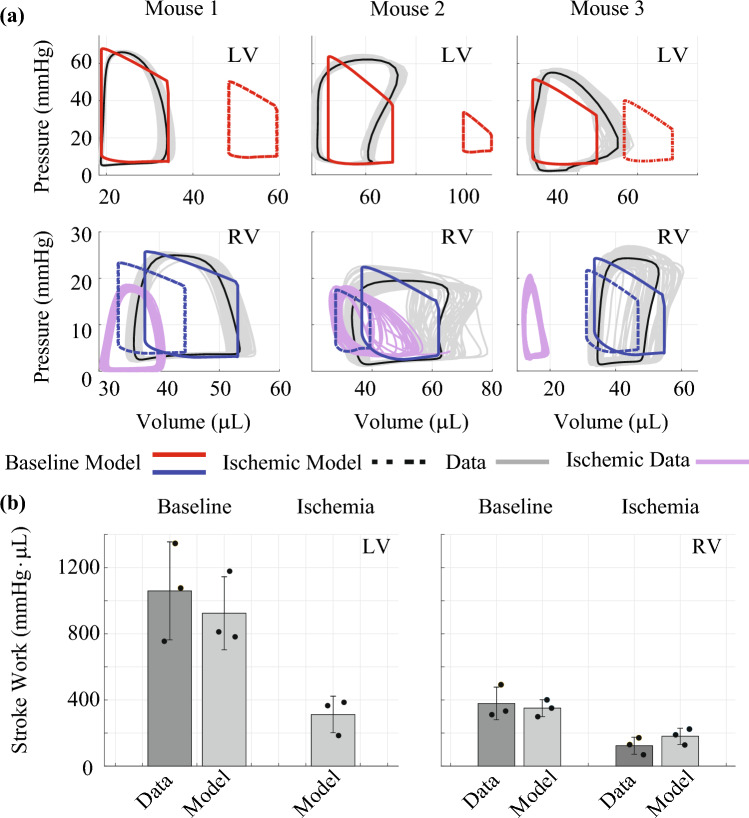
Changes in pressure–volume relationships with LV ischemia. **a** Baseline pressure–volume data (gray) in the LV (top) and RV (bottom) compared to the baseline simulations after parameter inference (solid, colored lines). Ischemic RV data (solid, blue) and RV predictions (dotted, blue). Note that LV ischemia causes a rightward shift in LV pressure–volume loops, while RV pressure–volume loops show a slight to moderate leftward shift with a reduction in stroke volume. Ischemic RV data vary with each mouse. **b** Ventricular stroke work (integral of the pressure–volume loop) at baseline and in ischemia. Stroke work is larger in the LV due to pressure magnitude, and both LV and RV stroke work are reduced in ischemia

We simulate ischemia by decreasing the LV active force generation using $\gamma^{\text{MI}}$. In-vivo changes in LV fractional shortening (equivalent to ejection fraction) in Fig. 2d show a 60%, 69%, and 54% reduction during ischemia, for mouse 1, 2, and 3, respectively. Simulated LV and RV outputs at baseline and in ischemia are shown in Fig. 5a. Using $\gamma_{ }^{\text{MI}} =$ 0.25, 0.15, and 0.25 reduced the ejection fraction by 58%, 71%, and 52%, for mouse 1, 2, and 3, respectively. Decreased contractile function shifts LV pressure–volume relationships rightward. Stroke volume in both heart chambers is reduced in ischemia, while diastolic RV pressure increases. Recorded RV pressure–volume data during ischemia also show a slight leftward shift as seen by the model. Data from mice 1 and 2 have a similar stroke volume to the model predictions, while mouse 3 has a substantial reduction in volume values. Ventricular stroke work, the area within the pressure–volume loop, is shown in Fig. 5b for both the data and model simulations. Stroke work is greater in the LV than the RV due to the difference in pressure magnitudes. Stroke work in both cardiac chambers decreases with LV ischemia.

**Fig. 6:**
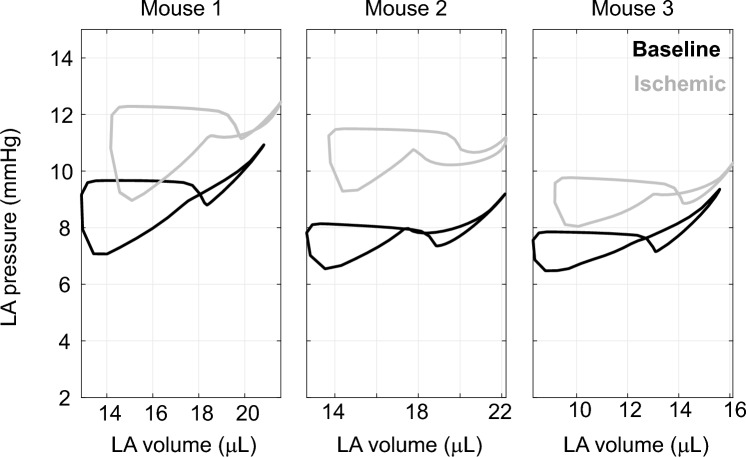
Left atrial (LA) pressure–volume loops from the model at baseline after parameter inference (black) and after LV ischemia (gray) in all three animals. The illustrated upward shift is indicative of elevated LV diastolic pressures. Note that baseline LA curves have the distinct “8” pattern seen in-vivo

We also investigate the systems-level effects of LV ischemia. Figure 6 displays left atrial pressure–volume loops at baseline and in ischemia. All three mice show an upward shift in ischemia, attributed to elevated LV diastolic and pericardial pressure (not shown). The latter increases on average 2 to 5 mmHg with ischemia.

**Fig. 7:**
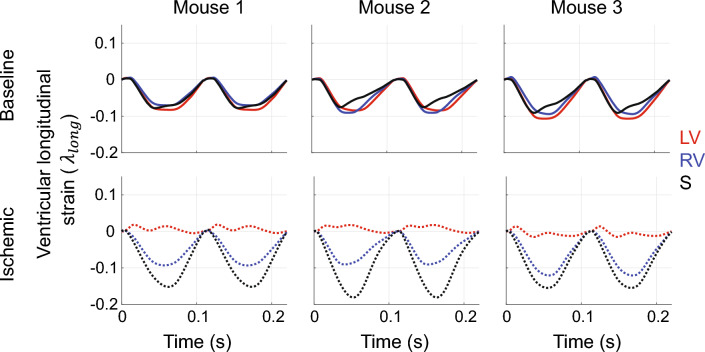
Predicted longitudinal strain in the LV, RV, and S in all three animals after parameter inference. At baseline, all three walls contract synchronously and reach 10% shortening. Ischemia reduces longitudinal strain in the LV, while RV strain is relatively unchanged and S strain is increased. Time to peak strain in the RV and S are more delayed in ischemia

Longitudinal strains for the LV, RV, and S at baseline and during ischemia are provided in Fig. 7. Strains for all three walls are in phase at baseline, indicative of synchronous muscle shortening. In contrast, ischemic LV longitudinal strains are less pronounced due to the inability for the heart chamber to contract. RV strains are relatively unchanged with ischemia, while S wall strain magnitude is higher in systole with ischemia.

**Fig. 8:**
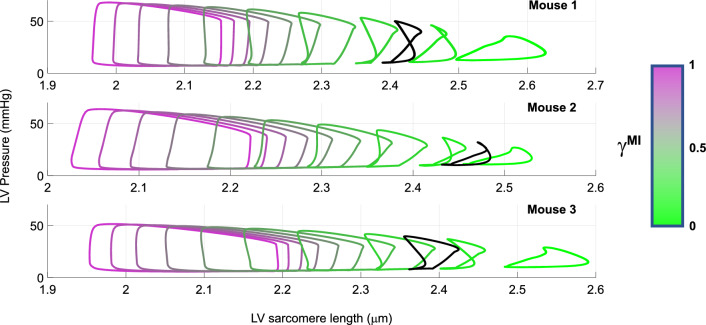
LV pressure–sarcomere length relationships in each mouse. Starting from each mouse’s optimal parameter set, the value of $\gamma^{\text{MI}}$ is decreased from 1.0 to 0.1, reflecting a 0–90% decrease in LV active force generation. The optimal value of $\gamma^{\text{MI}}$ is shown in black and provides a reduction in ejection fraction that best matches measurements in mice during ischemia. Note that the pressure–length curve has a distinct change in shape near the value of 0.2 and is qualitatively similar to prior studies using sonomicrometry [19]

To better understand the effects of decreased LV contractility, we show LV pressure versus sarcomere length for various values of $\gamma^{\text{MI}}$ in Fig. 8. Moving from baseline (magenta, far left) to nearly aberrant active force (green, far right), results show a decrease in LV pressure and elevated sarcomere lengths. The optimal degree of reduction for the data is plotted in black in Fig. 8. The pressure–length curves with larger reductions in active force exhibit a unique shape compared to less severe simulations.

## Discussion

This study combines multiscale computational modeling and parameter inference with in-vivo rodent data to investigate the acute biventricular consequences of LV myocardial ischemia. We identified a subset of cardiovascular parameters that can be made mouse-specific and calibrated them to hemodynamic data. We demonstrate that simulated acute LV ischemia raises septal wall strain, elevates left atrial pressure, and alters LV pressure–length relationships while RV function is relatively unchanged.

### Model Analysis

Multiscale models suffer from an imbalance in the number of model parameters versus available data. This inhibits inferring all system parameters and requires model analysis for robust parameter subsets. Morris screening is an efficient global sensitivity method to determine which parameters are non-influential, reduce simulation uncertainty, and enforce unique parameter values for each dataset [36]. Our screening results (Fig. 3) show that the reference areas ($A_{\text{m,ref}}$) and sarcomere contraction timing parameters ($\tau_{\text{rise,v}} ,\tau_{decay,v} ,$ and $\tau_{\text{sys,v}}$) are consistently influential. Van Osta et al. [23] performed a similar screening on their multiscale model of biventricular interaction. They identified reference areas, ventricular timing coefficients, and active force scaling factors as influential on ventricular wall.

We employed local sensitivity methods to further reduce our parameter space. Colunga et al. [6] used local sensitivity to reduce their parameter subset and ensure unique, unimodal posterior distributions for Bayesian inference. Their results showed that identifiability issues arose when using a non-influential subset of parameters. The parameter subset in the current study corroborates our previous findings [5]. However, the model used here accounts for pericardial constraints whereas our previous model did not. Computational studies by Pfaller et al. [25] and Sun et al. [31] have highlighted the importance of the pericardium on model predictions of LV and RV pressure. Sun et al. also showed that pericardial fluid volume altered hemodynamic predictions by upwards of 20%. This corroborates our findings that model predictions are sensitive to the reference pericardial volume, $V_{0,{\text{peri}}}$.

### Model Calibration

Identifying animal-specific parameters from in-vivo data can provide insight into unmeasurable properties of the cardiovascular system. Invasive measurements usually require multiple animals for sufficient evidence to test hypotheses; however, a combination of these data with mechanistic modeling can help investigate mechanisms of disease at a reduced animal burden. These models can also link data from multiple scales and multiple organs to highlight their underlying interactions, which is difficult to do with experiments alone.

Several previous studies have calibrated models to pressure–volume data in rodents. The study by Tewari et al. [32] calibrated model parameters to match data from mice subjected to 0, 14, 21, and 28 days of pulmonary arterial hypertension conditions (using chemical and environmental stimuli) [35]. The authors found an increase in $A_{\text{m,ref,RV}}$ and a decrease in $C_{\text{pa}}$ with increasing duration, which agrees with clinical understanding of RV and pulmonary adaptation in disease progression. Biventricular pressure–volume loop data have been recorded previously in rodents [11], yet this is the first study to use these data for model calibration. Results in Table 2 suggest that animals with larger end-diastolic volumes (e.g., Mouse 2) tend to have larger reference areas in that chamber. Our previous study [5] showed that model calibration to data from both ventricles reduced parameter and output uncertainty compared to only using RV data. The current study is one of the first to show that pressure–volume dynamics can be accurately captured in both ventricles using a biventricular heart model.

Calibrated model simulations shown in Fig. 4b are relatively consistent with systolic and diastolic pressure–volume data. The shapes of ventricular pressure curves are relatively well captured by our model; however, ventricular volume shapes are different between the model and the data. LV volume measurements recorded by Marquis et al. [20] were also more variable than the corresponding pressure measurements. Similar variability can be seen in RV volume recordings in the mouse study by Tewari et al. [32, 35]. Since there is inherent beat-to-beat variability in these measured quantities, we calibrate our model to heartbeat averaged signals.

Uncertainty quantification is a necessary, but often overlooked, step in the model analysis pipeline. Parameter confidence intervals, as determined in this work, provide substantially more information than point estimates alone. These analyses are warranted, especially as in-silico models gain traction as a potential bench side tool in the clinic. Here, we use asymptotic analyses based on frequentist statistical theory and incorporate heteroskedasticity by constructing error variance estimates for each measurement location [5, 20]. Model confidence intervals shown in Fig. 4b are narrower for the LV and RV pressure predictions in comparison to SA pressure and LV and RV volumes. This is linked to the high sensitivity of these states to the model parameters, as well as the larger mismatch between these data and the model. The wider prediction intervals contain most of data, with the largest prediction intervals associated with chamber volumes. The study by Marquis et al. [20] also provided output uncertainty in their model predictions using a similar methodology. However, our model prediction intervals are wider and contain a larger proportion of the data.

### Simulated Ischemia

Myocardial infarction is a precursor to long-term cardiac dysfunction and a risk factor for heart failure. There are multiple systems-level changes that occur during the onset of LV ischemia, which are critical in understanding survival rates and long-term cardiac remodeling [37]. A combined in-vivo and in-silico analysis can assist in understanding the interconnected dynamics of the cardiac chambers and vasculature in both physiological and pathological scenarios. Mechanistic models that tie together local and global cardiovascular function can be used to test hypotheses surrounding acute ischemic events and may be able to reduce the number of animals for in-vivo studies.

Data from all three mice show a reduction in fractional shortening and LV contractile function during ischemia. We simulate impaired LV function by reducing the active force generation by sarcomere shortening in the LV. Other authors have considered more sophisticated simulation strategies for ischemia. Witzenburg et al. [37] separated the LV into infarcted and non-infarcted regions, the latter only contributing to passive LV mechanics. Witzenburg also accounted for compensatory changes in afterload parameters and showed that model predictions matched well with prior experimental (canine) studies. Koopsen et al. [16] considered a similar, two-compartment approach for simulating LV infarction; these authors included biventricular interaction and showed agreement with previously obtained canine data from Lyseggen et al. [19].

Our simulated LV pressure–volume loops in Fig. 5a display a rightward shift with ischemia. Shiorua et al. [28] reported a similar shift in LV pressure–volume loops and a substantial (nearly 50%) reduction in LV stroke work two weeks after mice were subjected to LV ischemia. The ischemic RV simulations and data show a leftward shift in the pressure–volume loop, opposite to the LV. The reduced end-systolic and end-diastolic volumes are well captured in mice 1 and 2, whereas data from mouse 3 show a much larger shift in volumes after LV ischemia. Our agreement between the simulations and RV data during ischemia are attributed to the biventricular interaction model, as normal septal wall contraction prevents a large shift in blood volume away from the RV.

Experimentally, Damiano et al. [7] examined biventricular interaction by excising the sinoatrial node in mongrel dogs and controlling RV pacing. The authors noted that approximately 68% of RV systolic pressure was generated by the LV when ceasing RV pacing in dogs. Our model predicts a small decrease in RV systolic pressure in ischemia with relatively unchanged RV fractional shortening and pressure as measured by catheter. The discrepancy between Damiano’s finding and ours likely results from differences in experimental design (electrical pacing versus ligation), severity of the insult, species, and the unchanged dynamics of the shared septal wall.

Data from both ventricles enhance estimates of ventricular indices, including stroke work (Fig. 5b). Philip et al. [26] examined RV pressure–volume loops in mice eight weeks after LV ischemia and saw an increase in RV stroke work relative to the sham animals, which is contrary to our results. Philip et al. attributed this heightened stroke work to the development of pulmonary hypertension after ischemia, the severity of which depends on increased pulmonary vascular resistance. Since the increase in resistance is a chronic effect, the RV stroke work likely decreases at the onset of ischemia and then increases as pulmonary pressures rise due to pulmonary vascular remodeling [1].

The effects of myocardial infarction on LV systolic function are well studied, yet less work has focused on global changes during acute LV ischemia. Left atrial pressure–volume loops shift upward (Fig. 6) with the elevated LV end-diastolic pressure–volume relationship. Bauer et al. [3] reported a similar upward shift in bovine atrial pressure–volume loops during acute left anterior coronary artery occlusion. Another study by Hanif et al. [14] reported that mouse models of non-perfused myocardial infarction exhibit left atrial enlargement, atrial cardiomyocyte hypertrophy, and elevated left atrial fibrosis after multiple weeks. The elevation in left atrial pressure after LV ischemia is hypothesized to be a determinant of isolated post-capillary pulmonary hypertension [1]. Philip et al. [26] showed that LV ischemia in mice increases left atrial wall mass eight weeks after injury. Our model produces elevated pulmonary venous, left atrial, and pericardial pressures during acute LV ischemia, complementing these prior findings.

Strain results (Fig. 7) confirm equal levels of LV, RV, and S shortening at baseline. In ischemia, there is altered LV shortening and elevated S strain, with no apparent change in RV strain. Dann et al. [8] compared murine strain magnitudes after LV ischemia and reported significant reductions in LV free-wall shortening within the first seven days post ligation. Clinically, myocardial strain imaging is gaining traction as an indicator of heart function. Hamada-Harimura et al. [13] reported a strong correlation between RV free-wall longitudinal shortening and adverse cardiac events in acute decompensated heart failure suggesting that incompatible biventricular interactions might be indicative of mortality. The review by Smiseth et al. [29] identifies several novel uses for strain analysis in LV ischemia, especially during fibrosis and scar development. Further investigations into cardiac wall strain after ischemia are warranted, though our model simulations parallel the findings from the literature [16, 34]. The agreement in wall strains provides another validation point for our model and again highlights the importance of including the septum and biventricular interaction in model development.

As LV active force is reduced (i.e., as $\gamma^{\text{MI}}$ approaches zero), end-diastolic volumes and sarcomere lengths increase (Fig. 8). The shape of the pressure–length curve is maintained for initial reductions in LV active force but change for values of $\gamma^{\text{MI}}$ between 0.3 and 0.1. This “loop” like pattern was observed in Lyseggen et al. [19], who measured LV long-axis strain in canines during LV ischemia. The authors showed that the viable LV myocardial pressure–strain curve switched from counter-clockwise to clockwise after 15 minutes of ischemia. The recent computational study by Koopsen et al. [16] reproduced similar plots using a two-compartment model of the ischemic LV and simulated the effects of reperfusion that parallel results reported by Lyseggen et al. Our results, similar to Koopsen et al., illustrate how mechanistic, multiscale modeling can assist in verifying and simulating previously established findings in the literature.

### Limitations

Our study combines a multiscale model of cardiovascular dynamics with pressure–volume loop data from three animals. We plan to use a larger cohort of animals, including both male and female mice, in future studies. A larger amount of data would allow for proper characterization of the time-dependent pressure–volume relation, the signal biases, and possible corrections needed in pressure–volume loop shape. This would assist in determining the measurement error variances within the error covariance ${\hat{\Sigma }}$. We did not examine infarct size postmortem, though future studies could correlate these data with the degree of active force reduction, $\gamma^{\text{MI}}$. We simulate acute LV ischemia but do not account for any acute hemodynamic control mechanisms e.g., the baroreflex. These mechanisms play a role in the long-term homeostasis of the cardiovascular system [37], but it is unclear how quickly these response mechanisms act. Future studies across multiple days will require more detailed models of cardiovascular adaptation and remodeling. Detailed data on the RV response to LV ischemia are necessary; e.g., strain data on biventricular inefficiency and mechanical uncoupling (i.e., a transition from rightward to leftward septal motion) would provide information into the progression of RV dysfunction due to LV dysfunction.

We use a combination of global and local sensitivity analyses to reduce our parameter subset. More robust identifiability methods, such as profile-likelihood analyses [5], could be used to provide additional insight into parameter dependencies on the outputs. We construct estimates of both parameter and output uncertainty using asymptotic frequentist analyses. While this is a necessary first step, we make several assumptions in our statistical model. We assume that the measurement errors are independent, which is likely incorrect given the nature of both the pressure–volume catheter measurements and the underlying coupled physics in the ventricles. We also disregard model misspecification or model discrepancy, which requires a more rigorous mathematical and statistical analysis [17, 24]. Without details regarding measurement bias and precision, we likely overlook key components of the measurement error that could be informative when constructing our statistical model. Moving forward, more effort and research should be tailored toward understanding the underlying model and statistical assumptions, and the possible bias in parameter estimates that follow from these assumptions.

### Conclusions

We combine in-vivo biventricular pressure–volume loop data with a multiscale computational model of the cardiovascular system. Our study utilizes experimental data with multiscale modeling to identify parameter point estimates, their uncertainty, and the uncertainty in the model outputs. We present a framework that can benefit both the experimental and modeling communities as we move toward developing the field of digital health. Our results also show that LV and RV pressure–volume loops can be matched by the model. Our simulations of acute LV ischemia are in line with both recorded RV data and previously published studies documenting the LV’s response. This study displays systems-level hemodynamic changes during the acute stages of myocardial infarction and shows elevated left atrial pressures due to insufficient LV contraction. Our combination of in-vivo and in-silico techniques provides a framework for understanding the initial effects of LV ischemia and serves as a foundation for an improved understanding of cardiac and vascular remodeling in heart failure with reduced ejection fraction.

### Supplementary Information

Below is the link to the electronic supplementary material.

Supplementary file1 (PDF 1377 kb)

## References

[1] Allen B, Frye H, Ramanathan R, Caggiano LR, Tabima DM, Chesler NC, Philip JL (2023). Biomechanical and mechanobiological drivers of the transition from postcapillary pulmonary hypertension to combined pre−/postcapillary pulmonary hypertension. J. Am. Heart Assoc..

[2] Banks HT, Hu S, Thompson WC (2014). Modeling and Inverse Problems in the Presence of Uncertainty.

[3] Bauer F, Jones M, Jian XQ, Castro P, Asada J, Sitges M, Cardon LA, Tsujino H, Zetts AD, Panza JA, Thomas JD, Shiota T (2005). Quantitative analysis of left atrial function during left ventricular ischemia with and without left atrial ischemia: a real-time 3-dimensional echocardiographic study. J. Am. Soc. Echocardiogr..

[4] Burkhoff D, Tyberg J (1993). Why does pulmonary venous pressure rise after onset of LV dysfunction: a theoretical analysis. Am. J. Physiol. Heart Circ. Physiol..

[5] Colebank MJ, Chesler NC (2022). An in-silico analysis of experimental designs to study ventricular function: a focus on the right ventricle. PLoS Comput. Biol..

[6] Colunga AL, Kim KG, Woodall NP, Dardas TF, Gennari JH, Olufsen MS, Carlson BE (2020). Deep phenotyping of cardiac function in heart transplant patients using cardiovascular system models. J. Physiol..

[7] Damiano RJ, la Follette P, Cox JL, Lowe JE, Santamore WP (1991). Significant left ventricular contribution to right ventricular systolic function. Am. J. Physiol..

[8] Dann MM, Clark SQ, Trzaskalski NA, Earl CC, Schepers LE, Pulente SM, Lennord EN, Annamalai K, Gruber JM, Cox AD, Lorenzen-Schmidt I, Seymour R, Kim K-H, Goergen CJ, Mulvihill EE (2022). Quantification of murine myocardial infarct size using 2-D and 4-D high-frequency ultrasound. Am. J. Physiol..

[9] Dernellis JM, Stefanadis CI, Zacharoulis AA, Toutouzas PK (1998). Left atrial mechanical adaptation to long-standing hemodynamic loads based on pressure-volume relations. Am. J. Cardiol..

[10] Ellwein LM, Tran HT, Zapata C, Novak V, Olufsen MS (2008). Sensitivity analysis and model assessment: mathematical models for arterial blood flow and blood pressure. Cardiovasc. Eng..

[11] Faber MJ, Dalinghaus M, Lankhuizen IM, Steendijk P, Hop WC, Schoemaker RG, Duncker DJ, Lamers JMJ, Helbing WA (2006). Right and left ventricular function after chronic pulmonary artery banding in rats assessed with biventricular pressure-volume loops. Am. J. Physiol..

[12] Goldstein JA, Tweddell JS, Barzilai B, Yagi Y, Jaffe AS, Cox JL (1992). Importance of left ventricular function and systolic ventricular interaction to right ventricular performance during acute right heart ischemia. J. Am. Coll. Cardiol..

[13] Hamada-Harimura Y, Seo Y, Ishizu T, Nishi I, Machino-Ohtsuka T, Yamamoto M, Sugano A, Sato K, Sai S, Obara K, Yoshida I, Aonuma K (2018). Incremental prognostic value of right ventricular strain in patients with acute decompensated heart failure. Circ. Cardiovasc. Imaging.

[14] Hanif W, Alex L, Su Y, Shinde AV, Russo I, Li N, Frangogiannis NG (2017). Left atrial remodeling, hypertrophy, and fibrosis in mouse models of heart failure. Cardiovasc. Pathol..

[15] Jezek F, Randall EB, Carlson BE, Beard DA (2022). Systems analysis of the mechanisms governing the cardiovascular response to changes in posture and in peripheral demand during exercise. J. Mol. Cell. Cardiol..

[16] Koopsen T, Van Osta N, Van Loon T, Van Nieuwenhoven FA, Prinzen FW, Van Klarenbosch BR, Kirkels FP, Teske AJ, Vernooy K, Delhaas T, Lumens J (2022). A lumped two-compartment model for simulation of ventricular pump and tissue mechanics in ischemic heart disease. Front. Physiol..

[17] Lei CL, Ghosh S, Whittaker DG, Aboelkassem Y, Beattie KA, Cantwell CD, Delhaas T, Houston C, Novaes GM, Panfilov AV, Pathmanathan P, Riabiz M, Dos Santos RW, Walmsley J, Worden K, Mirams GR, Wilkinson RD (2020). Considering discrepancy when calibrating a mechanistic electrophysiology model: Discrepancy and mechanistic modelling. Philos. Trans. R. Soc. A.

[18] Lumens J, Delhaas T, Kirn B, Arts T (2009). Three-Wall Segment (TriSeg) Model describing mechanics and hemodynamics of ventricular interaction. Ann. Biomed. Eng..

[19] Lyseggen E, Skulstad H, Helle-Valle T, Vartdal T, Urheim S, Rabben SI, Opdahl A, Ihlen H, Smiseth OA (2005). Myocardial strain analysis in acute coronary occlusion: a tool to assess myocardial viability and reperfusion. Circulation.

[20] Marquis AD, Arnold A, Dean-Bernhoft C, Carlson BE, Olufsen MS (2018). Practical identifiability and uncertainty quantification of a pulsatile cardiovascular model. Math. Biosci..

[21] Marzban B, Lopez R, Beard DA (2020). Computational modeling of coupled energetics and mechanics in the rat ventricular myocardium. Physiome.

[22] Olsen CH, Ottesen JT, Smith RC, Olufsen MS (2019). Parameter subset selection techniques for problems in mathematical biology. Biol. Cybern..

[23] van Osta N, Lyon A, Kirkels F, Koopsen T, van Loon T, Cramer MJ, Teske AJ, Delhaas T, Huberts W, Lumens J (2020). Parameter subset reduction for patient-specific modelling of arrhythmogenic cardiomyopathy-related mutation carriers in the CircAdapt model. Philos. Trans. R. Soc. A.

[24] Paun LM, Colebank MJ, Olufsen MS, Hill NA, Husmeier D (2020). Assessing model mismatch and model selection in a Bayesian uncertainty quantification analysis of a fluid-dynamics model of pulmonary blood circulation. J. R. Soc. Interface.

[25] Pfaller MR, Hörmann JM, Weigl M, Nagler A, Chabiniok R, Bertoglio C, Wall WA (2018). The importance of the pericardium for cardiac biomechanics: from physiology to computational modeling. Biomech. Model Mechanobiol..

[26] Philip JL, Murphy TM, Schreier DA, Stevens S, Tabima DM, Albrecht M, Frump AL, Hacker TA, Lahm T, Chesler NC (2019). Pulmonary vascular mechanical consequences of ischemic heart failure and implications for right ventricular function. Am. J. Physiol..

[27] Rowson B, Duma SM, King MR, Efimov I, Saterbak A, Chesler NC (2021). Citation diversity statement in BMES journals. Ann. Biomed. Eng..

[28] Shioura KM, Geenen DL, Goldspink PH (2007). Assessment of cardiac function with the pressure-volume conductance system following myocardial infarction in mice. Am. J. Physiol. Heart Circ. Physiol..

[29] Smiseth OA, Torp H, Opdahl A, Haugaa KH, Urheim S (2016). Myocardial strain imaging: How useful is it in clinical decision making?. Eur. Heart J..

[30] Smith RC (2013). Uncertainty Quantification: Theory, Implementation, and Applications.

[31] Sun Y, Beshara M, Lucariello RJ, Chiaramida SA (1997). A comprehensive model for right-left heart interaction under the influence of pericardium and baroreflex. Am. J. Physiol. Heart Circ. Physiol..

[32] Tewari SG, Bugenhagen SM, Wang Z, Schreier DA, Carlson BE, Chesler NC, Beard DA (2013). Analysis of cardiovascular dynamics in pulmonary hypertensive C57BL6/J mice. Front. Physiol..

[33] Tsao, C. W. et al. Heart Disease and Stroke Statistics-2022 Update: A Report from the American Heart Association, 2022. 10.1161/CIR.0000000000001052 35078371

[34] Walmsley J, Arts T, Derval N, Bordachar P, Cochet H, Ploux S, Prinzen FW, Delhaas T, Lumens J (2015). Fast simulation of mechanical heterogeneity in the electrically asynchronous heart using the MultiPatch module. PLoS Comput. Biol..

[35] Wang Z, Schreier DA, Hacker TA, Chesler NC (2013). Progressive right ventricular functional and structural changes in a mouse model of pulmonary arterial hypertension. Physiol. Rep..

[36] Wentworth MT, Smith RC, Banks HT (2016). Parameter selection and verification techniques based on global sensitivity analysis illustrated for an HIV Model. SIAM/ASA J. Uncertain. Quantif..

[37] Witzenburg CM, Holmes JW (2019). The impact of hemodynamic reflex compensation following myocardial infarction on subsequent ventricular remodeling. J. Biomech. Eng..

[38] Zhou, D., E. Cornblath, J. Stiso, E. Teich, J. Dworkin, A. Blevins, and D. Bassett, Gender Diversity Statement and Code Notebook, 10.5281/zenodo.3672110

