# Supplementary material for: Biventricular Interaction During Acute Left Ventricular Ischemia in Mice: A Combined In-Vivo and In-Silico Approach
Source: Ann Biomed Eng. 2023 Jul 15;51(11):2528–43. doi: 10.1007/s10439-023-03293-z (PMC10598180; doi:10.1007/s10439-023-03293-z)
Supplement: Supplementary file 1 — Supplementary file1 (PDF 1377 kb) [file 10439_2023_3293_MOESM1_ESM.pdf]

Supplemental Material for: *Biventricular interaction during acute left ventricular ischemia in mice: a combined in-vivo and in-silico approach*

M. J. Colebank[1], R. Taylor[2], T. A. Hacker[2], N.C. Chesler[1,*]

[1]Edwards Lifesciences Foundation Cardiovascular Innovation and Research Center, and Department of Biomedical Engineering, University of California, Irvine, Irvine, CA, USA

[2]Cardiovascular Research Center, University of Wisconsin-Madison, Madison, WI, USA

* Corresponding author

Email: nchesler@uci.edu (NCC)

419 S. Circle View Drive, Suite 6800

Irvine, CA 92697

Blood volume and pressure estimates

Nominal parameters are generated using a combination of pressure and volume data or literature. Total blood volume in the mouse is determined by

$$V_{tot} = \mathrm{BW} \cdot 84.7 \frac{\mathrm{kg}}{\mathrm{ml}} \tag{S1}$$

as postulated by Riches et al.[7]. Bodyweight for mice 1, 2, and 3 were 29, 32.4, and 30.1 (g), respectively, giving V_{tot} values of 2.46, 2.74, and 2.55 mL. Average heart rates for mouse 1, 2, and 3 were 580, 495, and 532 (BPM), respectively. Stroke volume was determined from left (LV) and right ventricle (RV) volume measurements, set to be the

max of the two indices. Cardiac output (CO) was calculated as the product of stroke volume and heart rate. The assumed nominal pressures in the other cardiovascular compartment are calculated as a function of the LV and RV pressure data, as detailed in Table 1. The unstressed volumes (i.e., the blood not ejected during a cardiac cycle) and stressed volumes in each compartment are based on previous work[2] and provided in Table 2. The compartment pressures and stressed volumes are used to construct nominal estimates of vascular resistance and compliance, shown in Table 3.

Table 4 shows the calculated values of the Triseg geometry. Consistent units were necessary for multiscale interactions between model components, hence pressures were converted to KPa using the relation 1 mmHg = 0.133322 kPa, areas were converted from mm^2 to cm^2, and volumes were converted from μL to mL. Outputs from the model are subsequently scaled back for clarity in the results.

Cardiac Equations

The sarcomere model is embedded within a cardiac tissue model of atrial dynamics and biventricular interaction (the "TriSeg" model[4]). Changes in blood volume $V(t)$ (μl) cause distension in the cardiac chambers, giving rise to the myocardial strain ε_f

$$\varepsilon_f = \frac{1}{2}\ln\left(\frac{A_m}{A_{m,ref}}\right) - \frac{1}{12}z^2 - 0.019z^4\,,\ \ z = \frac{3C_m V_{wall}}{2A_m}. \tag{S2}$$

Here, A_m (cm^2) is the current mid-wall area of the chamber, $A_{m,ref}$ (cm^2) is the reference mid-wall area, and z (dimensionless) is a curvature variable related to the ratio of wall volume, V_{wall} (cm^3), and radius of mid-wall curvature C_m (cm^{-1}) [4]. Once ε_f

has been calculated and the corresponding G_{Tot} is obtained from the sarcomere model, the mid-wall tension can be calculated as

$$T_m = \frac{V_{wall} G_{Tot}}{2A_m}\left(1 + \frac{z^2}{3} + \frac{z^4}{5}\right). \quad \text{(S3)}$$

A balance in axial and radial tensions, T_x and T_y is enforced across the septal wall

$$\sum_{i=LV,RV,S} T_{x,i} = \sum_{i=LV,RV,S} T_{y,i} = 0 \quad \text{(S4)}$$

providing two differential algebraic equations [6]. The cavity tensions are used to calculate the cavity pressures.

The mid-wall volume, V_m (cm^3), mid-wall curvature, C_m (cm^{-1}), and mid-wall cross-sectional area, A_m (cm^2), are the driving variables for cardiac chamber dynamics. In the atria, these are described by

$$V_m = V(t) + \frac{1}{2} V_{wall}, \quad \text{(S5)}$$

$$C_m = \left(\frac{4\pi}{3V_m}\right)^{1/3}, \quad \text{(S6)}$$

$$A_m = \frac{4\pi}{C_m^2}. \quad \text{(S7)}$$

Since the LV, RV, and S are mechanically coupled, a separate formulation for V_m, C_m, and A_m is required (based on the TriSeg model[4]). Utilizing the common radius of mid-wall junction point y_m and denoting the maximal axial distance from each chamber wall surface to the origin as x_m[4], we get

$$V_m = \frac{\pi}{6} x_m (x_m^2 + 3y_m^2) \tag{S8}$$

$$C_m = \frac{2x_m}{(x_m^2 + y_m^2)}, \tag{S9}$$

$$A_m = \pi(x_m^2 + y_m^2), \tag{S10}$$

for the LV, RV, and S. We can also relate V_m to the blood volume $V(t)$ in the chamber

$$V_{m,LV} = -V_{LV}(t) - \frac{1}{2} V_{wall,LV} - \frac{1}{2} V_{wall,S} + V_{m,S} \tag{S11}$$

$$V_{m,RV} = -V_{RV}(t) + \frac{1}{2} V_{wall,LV} + \frac{1}{2} V_{wall,S} + V_{m,S}, \tag{S12}$$

which is updated at each time point.

The atrial transmural pressure is determined from the wall tension and mid-wall curvature

$$p = 2T_m C_m. \tag{S13}$$

The ventricular mid-wall tension is broken up into the axial (T_x) and radial (T_y) tensions based on the geometry of the spherical chambers and the angle of the sphere opening[4], giving

$$T_x = T_m \sin\left(\frac{2x_m y_m}{x_m^2 + y_m^2}\right), \quad T_y = T_m \cos\left(\frac{-x_m^2 + y_m^2}{x_m^2 + y_m^2}\right). \tag{S14}$$

These tensions must be balanced across the LV, RV, and septal wall, and serve as the algebraic constraints for the system as described above. The axial tensions in the ventricular cavities are then used to calculate the transmural pressure

$$p = \frac{2T_x}{y_m}. \tag{S15}$$

In total, the cardiac chambers and TriSeg model contribute two algebraic constraints (T_x and T_y being zero), five wall volume parameters (V_{wall}), and five reference area parameters ($A_{m,ref}$).

Heteroskedastic Errors and Asymptotic Uncertainty Quantification

We assume that the measurement errors in our data are independent and stem from a zero mean Gaussian distribution, i.e., $\varepsilon_{ij} \sim \mathcal{N}(0, \sigma_j^2)$, for each sequential data point $i = 1,2,\ldots,N$ and measurement source $j = 1,2,\ldots,5$. Our measurement sources include RV and LV pressure and volumes, as well as systemic arterial pressure. Parameter inference is performed on the natural-log scaled parameters using the negative log-likelihood, equivalent to minimizing

$$-LL(\boldsymbol{\theta}) = \frac{N}{2}\log(2\pi\ \det(\boldsymbol{\Sigma})) + \frac{1}{2}\left[\left(\boldsymbol{y}^{data} - f(\boldsymbol{t};\boldsymbol{\theta})\right)^{\top}\boldsymbol{\Sigma}^{-1}\left(\boldsymbol{y}^{data} - f(\boldsymbol{t};\boldsymbol{\theta})\right)\right] \tag{S16}$$

where $\boldsymbol{y}^{data}$ is the measured pressure and volume data, $f(\boldsymbol{t};\boldsymbol{\theta})$ is the corresponding simulations of the pressure and volumes. Under the assumption of independent errors, the covariance matrix, $\boldsymbol{\Sigma}$**,** is block diagonal

$$\boldsymbol{\Sigma} = \begin{bmatrix} \sigma_{RVP}^2\mathbf{I} & \mathbf{0} & \mathbf{0} & \mathbf{0} & \mathbf{0} \\ \mathbf{0} & \sigma_{RVV}^2\mathbf{I} & \mathbf{0} & \mathbf{0} & \mathbf{0} \\ \mathbf{0} & \mathbf{0} & \sigma_{LVP}^2\mathbf{I} & \mathbf{0} & \mathbf{0} \\ \mathbf{0} & \mathbf{0} & \mathbf{0} & \sigma_{LVV}^2\mathbf{I} & \mathbf{0} \\ \mathbf{0} & \mathbf{0} & \mathbf{0} & \mathbf{0} & \sigma_{SAP}^2\mathbf{I} \end{bmatrix} \tag{S17}$$

where the subscripts RVP, RVV, LVP, LVV, and SAP represent RV pressure, RV volume, LV pressure, LV volume, and systemic arterial pressure, respectively, and $\boldsymbol{I}$ is the identity matrix. Since the parameters, $\boldsymbol{\theta}$, and covariance matrix, $\boldsymbol{\Sigma}$, are coupled, we pursue a two-step updating scheme as follows[1]:

1. Using the initial guess for the natural-log scaled parameters, $\boldsymbol{\theta} = \boldsymbol{\theta}_{\mathbf{0}}$ and $\boldsymbol{\Sigma} = \mathbf{I}.$ This is equivalent to solving the ordinary least squares problem, which provides the current optimal value of the scaled parameter $\widehat{\boldsymbol{\theta}}.$
2. Calculate the residual vector $\hat{\boldsymbol{\varepsilon}} = \boldsymbol{y}^{data} - f(t; \widehat{\boldsymbol{\theta}})$ using the OLS estimate from step 1.
3. Set the error variance estimators as

$$\sigma_j^2 = \frac{1}{N_j} \sum_{i=1}^{N_j} \epsilon_{ij}^2 \tag{S18}$$

   where j denotes the measurement source and N_j is the number of samples associated with that measurement. In our work, $N_j = 50$ for each sample.

4. Set $\boldsymbol{\Sigma}$ using the results from equation (S17) in the block diagonal of (S18).
5. Minimize equation (S18) using $\widehat{\boldsymbol{\theta}}$ from the previous iteration and the updated covariance.
6. Repeat steps 2-5 until the scaled parameter vector has converged. We use the cutoff $\left(\widehat{\boldsymbol{\theta}}_{\iota} - \widehat{\boldsymbol{\theta}}_{i-1}\right)^2 \leq 1\mathrm{e} - 8$ to stop the algorithm.

Asymptotic uncertainty quantification is carried out using the final values of $\hat{\boldsymbol{\theta}}$ and $\hat{\boldsymbol{\Sigma}}$. As described in detail elsewhere[1,8], the statistical properties of the parameter estimate can be approximated using asymptotic theory, which gives rise to

$$\boldsymbol{\theta} \sim \mathcal{N}\left(\hat{\boldsymbol{\theta}}, \boldsymbol{C}\right) \quad \text{(S19)}$$

where the parameter variance-covariance estimator is approximated by

$$\boldsymbol{C} = \left(\hat{\boldsymbol{S}}^{\top}\hat{\boldsymbol{\Sigma}}^{-\mathbf{1}}\hat{\boldsymbol{S}}\right)^{-\mathbf{1}}. \quad \text{(S20)}$$

The term $\hat{\boldsymbol{S}}^{\top}\hat{\boldsymbol{\Sigma}}^{-\mathbf{1}}\hat{\boldsymbol{S}}$ approximates the Hessian matrix, $\frac{d^2LL}{d\boldsymbol{\theta}d\boldsymbol{\theta}^{\top}}$, which we calculate using sensitivity of the model outputs at the optimal parameter estimate in combination with the error covariance[1,8]. Using this approximation, the parameter confidence intervals can be constructed using a t-statistic and the parameter estimator variance-covariance matrix, giving

$$[\theta_i^-, \theta_i^+] = \hat{\theta}_i \pm t_{N_{tot}-N_{par}}^{0.975}\sqrt{\boldsymbol{C}_{ii}} \quad \text{(S21)}$$

where $N_{tot} = 250$ and $N_{par} = 11$ are the total number of data points and the number of parameters, respectively. The confidence and prediction intervals for the model output can be derived from the expected variance of the model response

$$\mathrm{Var}\big(f(t;\theta)\big) = \nabla f\ \mathrm{Var}\big(\hat{\boldsymbol{\theta}}\big)\ \nabla f^{\top} = \hat{\boldsymbol{S}}^{\top}\boldsymbol{C}\hat{\boldsymbol{S}} \quad \text{(S22)}$$

and the variance of a new, predicted value, $y^{new} = f(t;\theta) + \varepsilon_{new}$,

$$\mathrm{Var}(y^{new}) = \mathrm{Var}(\varepsilon_{new}) + \mathrm{Var}\big(f(t;\theta)\big) = \hat{\boldsymbol{\Sigma}} + \hat{\boldsymbol{S}}^{\top}\boldsymbol{C}\hat{\boldsymbol{S}}. \quad \text{(S23)}$$

The error variances for the three animals can be found in Table S1. The pairwise correlation between parameters at the inferred value is defined by $c_{ij} = C_{ij}/\sqrt{C_{ii}C_{jj}}$ and shown below in figure S1.

Table S1. Estimated noise variance, $\hat{\sigma}^2$, for the right ventricular pressure (RVP), right ventricular volume (RVV), left ventricular pressure (LVP), left ventricular volume (LVV), and systemic arterial pressure (SAP) in the three animals.

Animal	Mouse 1	Mouse 2	Mouse 3
$\hat{\sigma}^2{}_{RVP}$	4.7	2.6	3.0
$\hat{\sigma}^2{}_{RVV}$	2.2	34	24
$\hat{\sigma}^2{}_{LVP}$	13	30	18
$\hat{\sigma}^2{}_{LVV}$	1.2	28	11
$\hat{\sigma}^2{}_{SAP}$	7.7	32	30

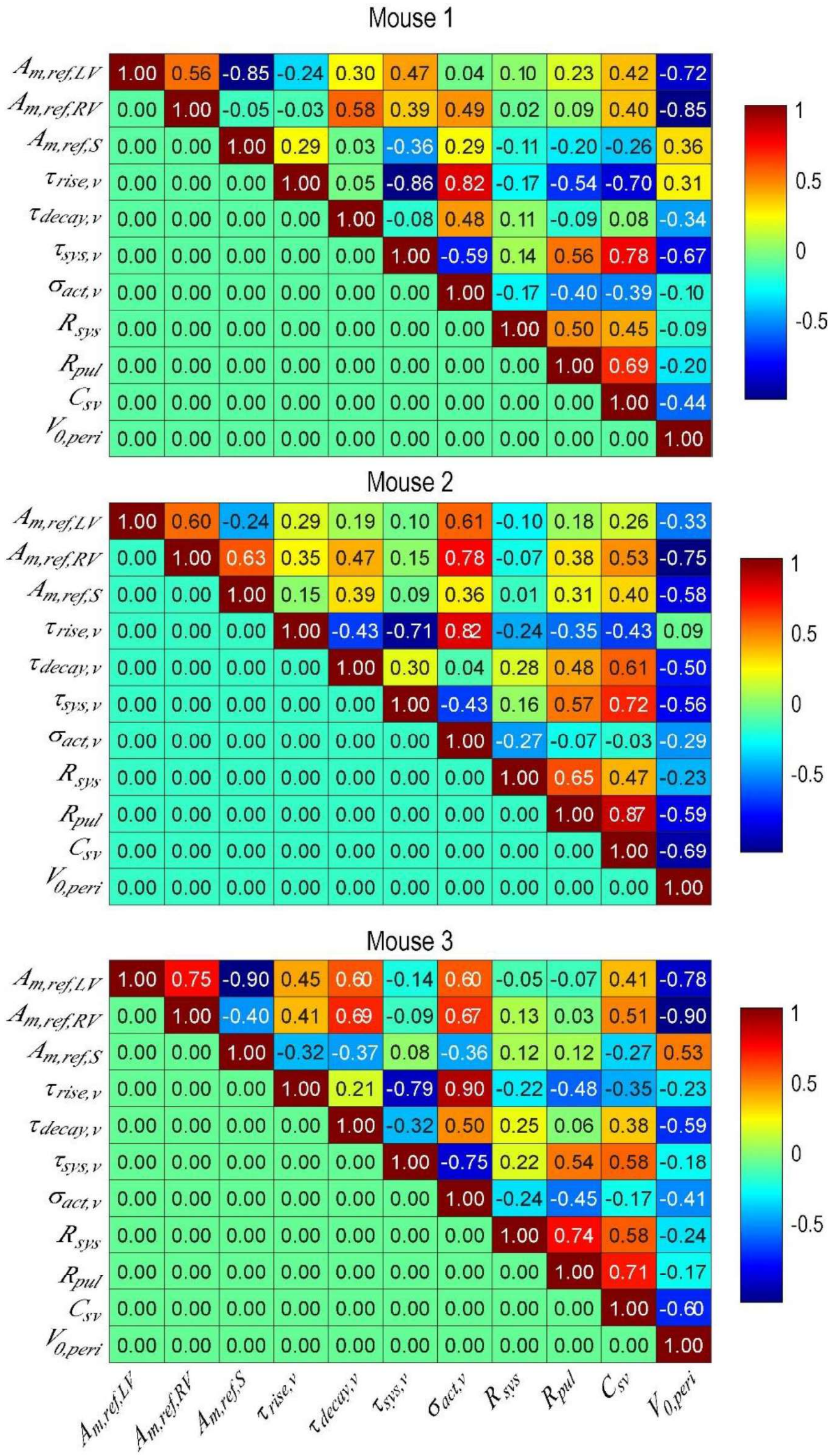

Fig. S1 Parameter correlations after optimization.

Table S2. Formulas for nominal pressure values (presented in mmHg for convenience). Data values are provided in brackets for mouse 1, 2, and 3, respectively.

Variable	Method/Value	Reference
$p_{LV,sys}$	[66, 62, 55]	Data
$p_{LV,dias}$	[5.2, 5.7, 2.1]	Data
$p_{RV,sys}$	[25, 20, 24]	Data
$p_{RV,dias}$	[2.5, 1.5, 1.4]	Data
$p_{LA,dias}$	$0.45 \cdot p_{pv,mean}$	Scaled from normotensive humans [3]
$p_{RA,dias}$	$0.45 \cdot p_{sv,mean}$	Scaled from normotensive humans [3]
$p_{sa,sys}$	$0.99 \cdot p_{LV,sys}$	Scaled from normotensive humans [3]
$p_{sa,dias}$	[52, 38, 30]	Data humans [3]
$p_{sa,mean}$	$(p_{sa,sys} + 2p_{sa,dias})/3$	Clinical definition [3]
$p_{pa,sys}$	$0.99 \cdot p_{RV,sys}$	Scaled from normotensive humans [3]
$p_{pa,dias}$	$0.32 \cdot p_{RV,sys}$	Scaled from normotensive humans [3]
$p_{pa,mean}$	$(p_{pa,sys} + 2p_{pa,dias})/3$	Clinical definition [3]
$p_{sys,cap}$	$0.26 \cdot p_{sa,mean}$	Scaled from normotensive humans [3]
$p_{pulm,cap}$	$0.70 \cdot p_{pa,mean}$	Scaled from normotensive humans [3]
$p_{sv,mean}$	$0.26 \cdot p_{sa,mean}$	Scaled from normotensive humans [3]
$p_{pv,mean}$	$0.15 \cdot p_{pa,mean}$	Scaled from normotensive humans [3]

Table S3. Formulas for nominal unstressed ($V_{i,un}$) and stressed volume (V_i) values (converted from μl to ml· 10^{-3}).

Variable	Method	Reference
$V_{sa,un}$	$0.06 \cdot V_{tot}$	Tuned from [3]
$V_{sv,un}$	$0.76 \cdot V_{tot}$	Based on [3]
$V_{RA,un}$	$0.01 \cdot V_{tot}$	Based on [3]
$V_{RV,un}$	$0.026 \cdot V_{tot}$	Based on [3]
$V_{pa,un}$	$0.027 \cdot V_{tot}$	Based on [3]
$V_{pv,un}$	$0.073 \cdot V_{tot}$	Based on [3]
$V_{LA,un}$	$0.010 \cdot V_{tot}$	Based on [3]
$V_{LV,un}$	$0.026 \cdot V_{tot}$	Based on [3]
V_{sa}	$0.27 \cdot V_{sa,un}$	Based on [2]
V_{sv}	$0.075 \cdot V_{sv,un}$	Based on [2]
V_{RA}	$1.0 \cdot V_{RA,un}$	Based on [2]
V_{RV}	$1.0 \cdot V_{RV,un}$	Based on [2]
V_{pa}	$0.58 \cdot V_{pa,un}$	Based on [2]
V_{pv}	$0.11 \cdot V_{pv,un}$	Based on [2]
V_{LA}	$1.0 \cdot V_{LA,un}$	Based on [2]
V_{LV}	$1.0 \cdot V_{LV,un}$	Based on [2]
$V_{0,peri}$	$0.9 \cdot (V_{RA} + V_{RV} + V_{LA} + V_{LV})$	Hand tuned
$V_{m,s}$	$0.0084 \cdot V_{tot}$	Hand tuned

Table S4. Nominal TriSeg geometry parameter values. Wall volumes (cm^3) are calculated as the ratio of excised chamber mass and myocardial density, assumed to be 1.053 g/ml. Reference areas (cm^2) are hand tuned initially to provide reasonable chamber volume predictions. All values are converted to cm in the model.

Parameter	Method/Value	Description	Reference
$V_{wall,LA}$	[2.85, 2.18, 3.89]$\times 10^{-3}$	Wall volume of left atrium	Based on[6]
$V_{wall,LV}$	[5.63, 7.67, 5.51] $\times 10^{-2}$	Wall volume of left ventricle	Based on[6]
$V_{wall,RA}$	[1.99, 3.89, 2.99]$\times 10^{-3}$	Wall volume of right atrium	Based on[6]
$V_{wall,RV}$	[1.99, 3.71, 1.67]$\times 10^{-2}$	Wall volume of right ventricle	Based on[6]
$V_{wall,S}$	[2.84, 3.84, 2.75]$\times 10^{-2}$	Wall volume of septum	Based on[6]
$A_{m,ref,LA}$	[0.15, 0.15, 0.15]	Reference mid-wall area of left atrium	Hand tuned
$A_{m,ref,LV}$	[0.55, 0.70, 0.55]	Reference mid-wall area of left ventricle	Hand tuned
$A_{m,ref,RA}$	[0.15, 0.15, 0.15]	Reference mid-wall area of right atrium	Hand tuned
$A_{m,ref,RV}$	[0.55, 0.75, 0.45]	Reference mid-wall area of right ventricle	Hand tuned
$A_{m,ref,S}$	[0.25, 0.25, 0.25]	Reference mid-wall area of septum	Hand tuned

Table S5. Formulas for nominal hemodynamic resistances (R, kPa·s/mL) and compliances (C, mL/kPa).

Parameter	Method/Value	Description	Reference
$R_{m,val}$	$\frac{0.1}{CO}$	Mitral valve resistance	Poiseuille/Ohm's Law
$R_{a,val}$	$\frac{0.75}{CO}$	Aortic valve resistance	Poiseuille/Ohm's Law
$R_{t,val}$	$\frac{0.1}{CO}$	Tricuspid valve resistance	Poiseuille/Ohm's Law
$R_{p,val}$	$\frac{0.75}{CO}$	Pulmonic valve resistance	Poiseuille/Ohm's Law
R_{vc}	$\frac{p_{sv,mean} - p_{ra,dias}}{CO}$	Vena Cava resistance	Poiseuille/Ohm's Law
R_{pv}	$\frac{p_{pv,mean} - p_{la,dias}}{CO}$	Pulmonary venous resistance	Poiseuille/Ohm's Law
R_{sys}	$\frac{p_{sa,sys} - p_{sys,cap}}{CO}$	Systemic vascular resistance	Poiseuille/Ohm's Law
R_{pulm}	$\frac{p_{pa,sys} - p_{pulm,cap}}{CO}$	Pulmonary vascular resistance	Poiseuille/Ohm's Law

C_{sa}	$\frac{V_{sa}}{p_{sa,sys}}$	Systemic arterial compliance	Based on [5]
C_{sv}	$\frac{V_{sv}}{p_{sv,mean}}$	Systemic venous compliance	Based on [5]
C_{pa}	$\frac{V_{pa}}{p_{pa,sys}}$	Pulmonary arterial compliance	Based on [5]
C_{pv}	$\frac{V_{pv}}{p_{pv,mean}}$	Pulmonary venous compliance	Based on [5]

References

1. Banks, H. T., S. Hu, and W. C. Thompson. Modeling and Inverse Problems in the Presence of Uncertainty. Chapman and Hall/CRC, 2014.doi:10.1201/b16760
2. Beneken, J. E. W., and B. DeWit. A physical approach to hemodynamic aspects of the human cardiovascular system. In: Physical Bases of Circulatory Transport: Regulation and Exchange., edited by A. Guyton, and E. Reeve. Philadelphia: Saunders, 1966, pp. 1–45.
3. Boron, W. F., and E. L. Boulpaep. Medical physiology. Philadelphia, PA : Elsevier, [2017], 2017.
4. Lumens, J., T. Delhaas, B. Kirn, and T. Arts. Three-Wall Segment (TriSeg) Model Describing Mechanics and Hemodynamics of Ventricular Interaction. *Ann Biomed Eng* 37:2234–2255, 2009.
5. Marquis, A. D., A. Arnold, C. Dean-Bernhoft, B. E. Carlson, and M. S. Olufsen. Practical identifiability and uncertainty quantification of a pulsatile cardiovascular model. *Math Biosci* 304:9–24, 2018.
6. Marzban, B., R. Lopez, and D. A. Beard. Computational Modeling of Coupled Energetics and Mechanics in the Rat Ventricular Myocardium. *Physiome* , 2020.doi:10.36903/physiome.12964970
7. Riches, A. C., J. G. Sharp, D. B. Thomas, and S. V. Smith. Blood volume determination in the mouse. *J Physiol* 228:279–284, 1973.
8. Smith, Ralph, C. Uncertainty Quantification: Theory, Implementation, and Applications. Society of Industrial and Applied Mathematics, 2013.
